# Acceptability and performance of dual HIV/syphilis testing in male circumcision clients, 2021

**DOI:** 10.4102/sajhivmed.v25i1.1571

**Published:** 2024-08-30

**Authors:** Tendesayi Kufa, Ocean Tobaiwa, Ewaldé Cutler, Beverley Singh, Zinhle Brukwe, Venessa Maseko, Erushka Pillay, Philip Dorrell, Khumbulani Moyo, Lindokuhle Zondi, Yogan Pillay, Sean Patrick, Adrian Puren

**Affiliations:** 1 Centre for HIV and Sexually Transmitted Infections, National Institute for Communicable Diseases, Johannesburg, South Africa; 2 School of Public Health, Faculty of Medicine, University of the Witwatersrand, Johannesburg, South Africa; 3 Clinton Health Access Initiative, Pretoria, South Africa; 4 Right to Care, Centurion, South Africa; 5 School of Health Systems and Public Health, Faculty of Health Sciences, University of Pretoria, Pretoria, South Africa; 6 Department of Virology, School of Pathology, Faculty of Medicine, University of the Witwatersrand, Johannesburg, South Africa

**Keywords:** male circumcision, HIV, syphilis, dual testing, acceptability, performance

## Abstract

**Background:**

Dual HIV/syphilis testing may be an acceptable intervention to identify men with sexually transmitted infections (STIs) and at risk of HIV acquisition.

**Objectives:**

We sought to determine the acceptability, and performance of dual HIV/syphilis testing among men attending voluntary medical male circumcision (VMMC) services at six public sector facilities in Gauteng.

**Method:**

This was a cross-sectional study at VMMC facilities. Men ≥ 18 years were enrolled. The men had (1) a questionnaire administered, (2) on-site dual HIV/syphilis testing with First Response HIV1+2/Syphilis Combo Card Test by routine lay counsellors, and (3) a blood specimen collected for centralised laboratory testing for HIV and syphilis serology. We evaluated pre-test and post-test acceptability and performance compared to serological testing.

**Results:**

Of the 679 men analysed (median age 32.1 years), 96.7% of HIV-negative men preferred testing for HIV and syphilis simultaneously. Of the 675 men tested for syphilis, 28 (4.7%) tested positive (past or recent). In the laboratory, 43/609 (7.1%) had syphilis infection detected, with 9/609 (1.5%) having recent syphilis. There was sub-optimal sensitivity for HIV detection (90.9%; 95% confidence interval [CI]: 88.5% – 93.3%), and for past/recent syphilis (55.8%; 95% CI: 51.9% – 59.8%), improving to 88.9% (95% CI: 86.4% – 91.4%) for recent syphilis. Specificities were > 99% for HIV and syphilis (past or recent). Post-test acceptability was 96.6% and willingness to pay for future testing was 86.1%.

**Conclusion:**

Dual HIV/syphilis testing was acceptable but had sub-optimal sensitivity for HIV and syphilis. Syphilis detection was adequate for recent infection.

**What this study adds:** This study demonstrates that dual HIV/syphilis screening was feasible and acceptable to men attending VMMC services but had sub-optimal sensitivity. Offering syphilis testing and other STI screening within VMMC services may attract older men at risk of HIV and STIs to these services.

## Introduction

South Africa has a large burden of sexually transmitted infections (STIs) other than HIV. In 2017, there were 23 000 new syphilis cases among women aged 15–49 years, and 47 500 among men of the same age.^1^ Syphilis can lead to complications and relationship conflict, which may contribute to gender-based violence in the country.

STIs are known to increase the risk of HIV acquisition and predict later seroconversion.^2,3^ Syphilis has been considered a harbinger for HIV seroconversion in studies among men who have sex with men (MSM).^3^ Among cis-gender men who were circumcised, evidence of recent syphilis infection and past infection with herpes were associated with HIV positivity among men attending STI services.^4^ This is concerning given that the prevalence of recent syphilis infection among pregnant women attending public health clinics has increased from 1.6% in 2011, to 2% in 2015, and 2.6% in 2019.^5^ In addition, there has been a steady increase in congenital syphilis cases reported to the National Institute for Communicable Diseases (NICD).^6^ These increases suggest increased transmission of syphilis in the general population, including men. Multiple factors are likely behind this observed increase in syphilis transmission and include low condom use,^7^ and shortages of benzathine penicillin in the country,^8^ resulting in the growing use of doxycycline with concerns around sub-optimal adherence and incomplete treatment.^9^

The burden of syphilis among men in South Africa has not been well described. Modelling work in South Africa estimates that the incidence and prevalence of syphilis among men were almost twice as high as women.^1^ Screening and treating for syphilis among men could be an effective, yet under-researched, strategy to reduce the burden of syphilis in the general population and reduce the risk of HIV transmission or acquisition among older men. Syphilis screening could also be a useful tool to increase traffic to voluntary medical male circumcision (VMMC) services and increase demand for VMMC. A dual HIV/syphilis test that allows for screening of HIV and syphilis at the same could be an appropriate tool to achieve this.

At least seven dual HIV/syphilis tests have been developed and are commercially available and three are prequalified by the World Health Organization (WHO).^10,11^ Of these, the First Response HIV1+2/Syphilis Combo Card Test was the first to be prequalified by the WHO in 2019,^12^ and has been used in laboratory and field evaluations, clinical settings and surveys since then, with sensitivities and specificities of 80% – 100%.^13,14,15^ The National Department of Health (NDOH) in South Africa started introducing rapid dual HIV/syphilis testing for pregnant women in response to rising syphilis rates among pregnant women, the need for more frequent testing during pregnancy, and to delays in starting appropriate treatment that was associated with laboratory testing.^5^ The NDOH is considering expanding rapid dual HIV/syphilis testing to other priority populations with respect to STIs and syphilis – MSM, transgender women (TGW), female sex workers (FSW) and people receiving pre-exposure prophylaxis. We evaluated the acceptability, outcomes and performance of a dual HIV/syphilis test among men attending VMMC services at six public sector facilities located in two districts of Gauteng Province, South Africa.

## Research methods and design

### Study design

The study used a cross-sectional design.

### Setting

This study was conducted in six high-volume VMMC clinics operated by the NDOH in partnership with a local non-governmental organisation, Right to Care (RTC).^16^ During 2020–2021, RTC supported VMMC sites in three districts of Gauteng Province.^16^ Men attending VMMC facilities were routinely offered STI symptom screening, as well as HIV counselling and testing and, if they had no specific contraindications for medical circumcision, they would be circumcised. Men with genital discharge or ulcers were offered treatment according to the current syndromic management guidelines and asked to return to VMMC once the symptoms had resolved.^16^

### Study population

Men ≥ 18 years attending VMMC services at selected high-volume VMMC sites, regardless of the reason for attendance, were eligible for inclusion. Men who were unable or unwilling to provide written informed consent and those who were acutely ill were ineligible for enrolment.

### Data collection

Following eligibility assessment, informed consent and enrolment procedures, a health provider administered a questionnaire to willing and eligible men. The questionnaire included questions collecting demographic information, clinical history, sexual behavioural and risk assessment, knowledge and perception about the dual HIV/syphilis rapid test. Some questions were adopted from the generic WHO protocol for evaluating rapid dual HIV/syphilis tests.^17^ The questionnaire also included the three questions from the NDOH STI symptom screening tool (Online Appendix 1 Table 1).^18^ The questionnaire was available in both electronic (Qualtrics® XM survey software, Seattle, WA, United States) and paper-based formats. Pre-test counselling for both the HIV and syphilis infections was provided to those enrolled. For HIV infection, this was conducted according to the NDOH HIV counselling and testing guidelines.^19^ For syphilis, men were counselled on the meaning of a positive rapid syphilis test and the need for follow-up confirmatory testing with the rapid plasma reagin (RPR) test. If the RPR was positive, it indicated recent syphilis infection which may have required treatment. Finger-prick blood specimens for rapid syphilis or rapid dual HIV/syphilis testing were collected from men who consented to testing and results were captured on the questionnaire. Enrolled men were also asked to provide 8.5 mL of blood for screening and confirmatory HIV testing, and full syphilis testing using the reverse testing algorithm. This algorithm uses a specific treponemal test (TPab) and a screening test and a non-specific treponemal test (RPR) as confirmatory, unlike the traditional algorithm which uses a non-treponemal test as screening and a treponemal test as confirmatory.^20^ Clients who tested positive for HIV on site were referred for HIV care, including antitretroviral therapy (ART). Those who tested positive for syphilis on the rapid dual HIV/syphilis test were provided with treatment on site and assessed for PrEP eligibility (where available) or were referred to the nearest primary care service for treatment and assessment for pre-exposure prophylaxis (PrEP) eligibility and initiation. Participants who had positive results for syphilis were treated for latent syphilis according to the NDOH STI treatment guidelines which, at the time, recommended three one-weekly intramuscular doses of benzathine penicillin or 100 mg of doxycycline twice a day for 28 days. If laboratory testing indicated previous infection and no active infection, the clients were meant to be called to stop treatment.

### On-site and laboratory testing

On-site HIV/syphilis testing was conducted using the First Response HIV1+2/Syphilis Combo Card Test (Premier Medical Corporation Pvt Ltd, Gujarat, India). The test was a rapid, qualitative screening, in-vitro diagnostic test for the detection of antibodies (IgG & IgM) specific to HIV (types 1 and 2) and *Treponema pallidum* in human serum, plasma or venous and capillary whole blood.^12^ The test was WHO-prequalified with in-vitro sensitivity and specificity for each analyte of ≥ 99%. The time to result was set at 20 minutes and positive results were meant to be confirmed in the laboratory using treponemal or non-treponemal assays.^21^ For men known to be HIV positive at enrolment, the single syphilis test version was used – First Response Syphilis Anti-TP Card Test (Premier Medical Corporation Pvt Ltd, Gujarat, India).^12^ On-site testing was conducted by trained lay counsellors according to manufacturer’s specifications and study-specific standard operating procedures (SOPs).

Results were documented on a study-specific testing register along with the participant’s study-generated ID number, date of test, and batch and lot numbers of the test used. The results were recorded on the online questionnaire and on the specimen request form sent to the laboratory along with the blood specimen.

All individuals who were enrolled and provided a blood specimen were tested for HIV using two 4th-generation HIV enzyme immunoassays (EIAs) in the HIV Sero-Molecular Reference Laboratory housed at the Centre for HIV and STIs (NICD). Initial screening used the Architect HIV Ag/Ab Combo (Abbott, Wiesbaden, Germany) with Genscreen Ultra HIV Ag/Ab (Bio-Rad, Marnes-la-Coquette, France) used for confirmatory testing. Syphilis testing in the STI reference laboratory, also housed at the Centre for HIV and STIs (NICD), was conducted using the reverse testing algorithm. This algorithm screened for specific treponemal antibodies using the Architect Syphilis *Treponema pallidum* antibody assay (TPAb) (Abbott, Wiesbaden, Germany) and then confirmed with the non-specific test – RPR test (Immutrep; Omega Diagnostics Ltd, Alva, United Kingdom) if the specimen was positive on TPAb. Specimens that were positive on RPR – indicating a recent infection – had antibody titre levels measured. Titres ≥ 1:8 were considered to be indicative of active and untreated syphilis infection. Titres of 1:1 to < 1:8 were considered to have had previous treatment and were serological non-responders.^20^ All samples were collected according to the SOPs for proper collection, processing, labelling, and transport of specimens to the laboratories. The specimens were tested according to the reference laboratory SOPs. Leftover specimens were discarded at the end of the study.

### Data management

Data collection was electronic. Study questionnaires were accessible to study staff on password-protected electronic tablets. The data were captured in a live Qualtrics® XM database that was secure and housed by the University of Pretoria. When internet connectivity was lost, healthcare providers completed the paper-based format of the questionnaire and captured the data later, when connectivity was restored. Data were extracted periodically to check for quality and consistency of completion. Data collection was within the framework of the University of Pretoria Code of Ethics for Research (Document Rt 429/99). At the end of the study, de-identified data were extracted in comma-separated files and exported into Stata 18.0 (Stata Corporation, College Station, TX, United States) for analysis. Laboratory results were reported on the NICD laboratory information system (LIMS), TrakCare (InterSystems®,Cambridge, MA, United States) and extracted as Excel files for analysis.

### Sample size calculations

The minimum sample size was determined to be 1080 across all the VMMC facilities. This sample size was calculated assuming a recent syphilis prevalence of 1% – 2%, 10% of enrolled would refuse testing or would be ineligible for testing after the initial consent for enrolment, an α- level of 5%, and 80% power. Assuming that the six high-volume VMMC sites would enrol to the same extent, each VMMC site was meant to enrol 200 men over 2 months. Both sample size calculations and the determination of number of facilities assumed that the prevalence of recent syphilis was the same across the VMMC sites.

### Statistical analysis and description of outcomes

The enrolled men were described using frequencies and proportions for categorical data, medians and interquartile ranges (IQRs) for continuous variables. The acceptability of dual HIV/syphilis testing was determined from responses on pre- and post-test scales. The post-test acceptability scale was a Likert scale with five items. For each person, individual item scores were summed up to generate a total score on the scale. Acceptability was defined as the proportion who scored highly on the acceptability scores. The outcomes of HIV and syphilis testing were the percentage of those tested on site and in the centralised laboratory who tested positive for HIV, syphilis alone, or both HIV and syphilis. The performance of rapid testing against laboratory-based serological testing as reference was determined in terms of sensitivity, specificity, positive and negative predictive values with overall agreement between on-site testing and centralised laboratory testing, and presented as proportions with 95% confidence intervals (CI). Performance was also determined by facility.

### Ethical considerations

The protocol for the study was submitted to the University of Pretoria Health Research Ethics Committee for ethical approval (Ethics number 735/2020). Approvals were also sought from the Gauteng Department of Health and the Gauteng Provincial Ethics Committee. Written informed consent for completion of questionnaires, dual HIV/syphilis testing and collection of a blood sample was obtained from each participant at enrolment. Participants were informed that their participation in the study was voluntary, and that they could withdraw consent at any time without any consequence to themselves or the care they sought. Participants who agreed to participate in the study were assigned a unique study ID number generated at enrolment, but were also asked to provide their contact details so that healthcare providers could phone them with results from the laboratory. Participants whose rapid HIV or syphilis and laboratory results were discrepant were called back for retest. The contact details were kept in a lockable cupboard, with access restricted to study staff.

## Results

### Demographic and behavioural characteristics of the enrolled men

Between 03 June 2021 and 08 October 2021, 749 men were enrolled at six VMMC facilities in the two districts. Because of requirements to comply with end-of-grant requirements, enrolments could not continue beyond 08 October 2021 and so the targeted sample size could not be achieved.

After excluding individuals with duplicate enrolments (11/70), missing participant ID numbers (4/70) and incomplete consent forms (55/70), there were 679 unique men included in the analysis. Table 1 describes the demographic, clinical and behavioural characteristics of these men. The median age was 32.1 years (IQR 25.8 – 38.5 years). Most men were enrolled in Tshwane District (82.5%) with a minority enrolled in Ekurhuleni (17.5%). Most men had completed some or all secondary education (80.4%), were unemployed or looking for work (39.7%), and had never been married (62.6%). The majority of men reported ever having sex (97.6%), with a median age at sexual debut of 17 years (16–19 years). Of the men who reported ever having sex, a large majority (98.6%) reported having sex with women only in the past 12 months. A significant proportion of the enrolled men reported sexual behaviour associated with HIV transmission in the months preceding enrolment – not using a condom at the last sexual encounter (59.8%), having sex under the influence of drugs and/or alcohol (18.6%), having sex with two or more partners (18.2%), and paying for sex (15.4%). The majority of men knew their HIV status (76.8%), with 12.7% reporting being HIV positive and 93.7% of them taking antiretroviral drugs (ARVs) in the 3 days preceding enrolment.

**TABLE 1:** Demographic, behavioural and clinical characteristics of the enrolled men (*N* = 679).

Category	Characteristics	*N*	*n*	%	Median	IQR
Demographic	**District**	679	-	-	-	-
	Ekurhuleni	-	119	17.5	-	-
	Tshwane	-	560	82.5	-	-
	**Clinic**	679	-	-	-	-
	Ekurhuleni 1	-	81	11.9	-	-
	Ekurhuleni 2	-	17	2.5	-	-
	Ekurhuleni 3	-	21	3.1	-	-
	Tshwane 1	-	183	27.0	-	-
	Tshwane 2	-	184	27.1	-	-
	Tshwane 3	-	193	28.4	-	-
	Age in years	679	-	-	32.1	25.8–38.5
	South African born†	677	569	84.1	-	-
	**Years of schooling completed**	679	-			
	Primary (0–7)	-	65	9.6	-	-
	Secondary (8–12)	-	546	80.4	-	-
	Tertiary (Diploma/degree/postgraduate studies)	-	62	9.1	-	-
	Other	-	6	0.9	-	-
	**Marital status**	671	-	-	-	-
	Never married	-	420	62.6	-	-
	Married/living as married	-	220	32.8	-	-
	Divorced/widowed	-	31	4.6	-	-
	**Employment status**	668	-	-	-	-
	Formally employed (part-time/full-time)	-	247	37.0	-	-
	Self-employed	-	82	12.3	-	-
	Student/Learner	-	64	9.6	-	-
	Unemployed/looking for work	-	265	39.7	-	-
	Unable to work	-	10	1.5	-	-
Behavioural	Ever had sex	679	663	97.6	-	-
	Age at first sex	627	-	-	17.0	16.0–19.0
	Sex with women only in the past 12 months	640	631	98.6	-	-
	Ever paid for sex	662	102	15.4	-	-
	Ever been paid to have sex	638	26	4.1	-	-
	Sex under the influence of alcohol or drugs in the past 3 months	635	118	18.6	-	-
	Sex with a partner living in a different province in the past 3 months	639	44	6.9	-	-
	Sex with a partner living in a different country in the past 3 months	616	41	6.1	-	-
	Condom use at last sexual encounter	625	251	40.2	-	-
	Number of sexual partners in the last 3 months	434	-	-	1.0	0.0–4.0
	Two or more sexual partners in the past 3 months	434	79	18.2	-	-
	Considers themselves at risk of getting HIV	580	119	20.5	-	-
Clinical	Treated for an STI in the past 12 months	525	18	3.4	-	-
	Genital discharge or ulcers present at enrolment	663	14	2.1	-	-
	Know HIV status	650	499	76.8	-	-
	HIV positive at most recent HIV test	497	63	12.7	-	-
	Ever taken ART for own health	63	61	96.8	-	-
	Taken ARVs in the past 3 days	63	59	93.7	-	-

ART, antiretroviral therapy; ARV, antiretroviral; IQR, interquartile range; STI, sexually transmitted infection.

† , Of the 108 not born in South Africa, 58 (53.7%) were born in Zimbabwe, 30 (28.8%) in Mozambique, 14 (13.0%) in Malawi, 3 in Swaziland, 2 in Lesotho, and 1 in Uganda.

### Pre-test acceptability of HIV and syphilis testing

The overwhelming majority of men wanted to test for syphilis (99.9%), with slightly fewer wanting to test for HIV (97.9% among those not known to be HIV positive) – Table 2. Most men wanted to test for HIV and syphilis simultaneously (96.7%). These men preferred the dual HIV/syphilis test to two single tests (95.3% vs 4.1%), largely because they wanted to know their HIV and syphilis status and because the dual HIV/syphilis test would be convenient and quick. Most men were willing to wait for their results for up to 20 min, which was the recommended waiting time for the assay.

**TABLE 2:** Pre-test acceptability of HIV and syphilis testing among enrolled men (*N* = 679).

Item	*N*	*n*	%
Responded yes to ‘Would you like to be tested for HIV?’	611†	598	97.9
Responded yes to ‘Would you like to be tested for syphilis?’	672	671	99.9
Responded yes to ‘Would you like to be tested for HIV and syphilis at the same time?’	613†	593	96.7
**Preferred two single tests or one combined**	613†	-	-
One combined (dual test)	-	584	95.3
Two single tests	-	25	4.1
I do not know	-	3	0.5
It is the same	-	1	0.2
**Reasons for preferring combined tests**	599†	-	-
Wants to know both syphilis and HIV results/status	-	388	64.8
Convenient (saves time, quick, easy)	-	201	33.6
Other	-	7	1.2
No response	-	3	0.5
**Reasons for not preferring combined tests**	14†	-	-
Already knows HIV status	-	6	42.9
Don’t want to know HIV status	-	6	42.9
Single tests maybe more accurate	-	1	7.1
Just want two tests	-	1	7.1
Responded yes to ‘Would you be willing to wait for the results at the clinic directly after the tests are performed?’	672	647	96.3
**Response to ‘How long will you willing to wait for your results?’**	646	-	-
Up to 20 min	-	531	82.2
Up to 30 min	-	105	16.3
Up to 1 h	-	2	0.3
Up to 2 h	-	1	0.2
I don’t know	-	7	1.1

† , Among those who did not self-report being HIV positive.

### Outcomes of HIV and syphilis testing

**FIGURE 1:**
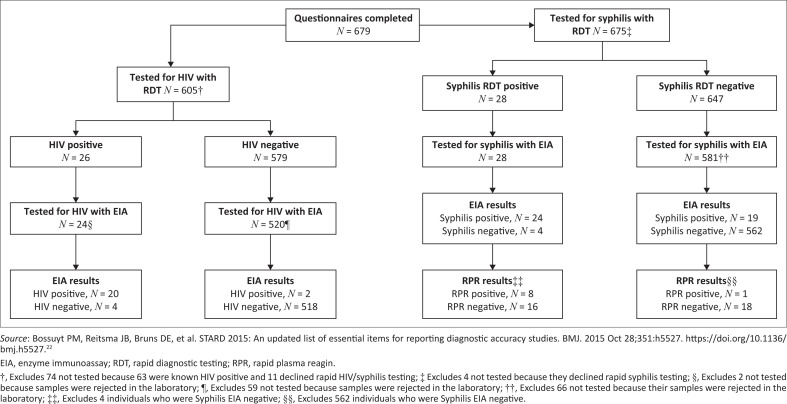
Yield of HIV and syphilis testing.

Figure 1 describes the outcomes of on-site rapid diagnostic testing (RDT) and centralised laboratory testing according to the guidelines for reporting studies of diagnostic test performance.^26^ Of the 679 men who completed a questionnaire, 605 (89.1%) were tested for HIV using the RDTs. This excluded 63 men who self-reported being HIV positive and were therefore not eligible for HIV testing, as well as 11 men who did not want to be tested for HIV. Of the 605 men who were tested using the dual HIV/syphilis RDT, 26 (4.3%) tested positive for HIV infection, with 20 (83.3%) of the RDT positives further confirmed as HIV positive on EIA testing in the laboratory. In total, 22/544 (4.0%) men tested for HIV in the centralised laboratory had previously undiagnosed HIV.

For syphilis, 675 men tested on site using either the single or dual HIV/syphilis RDT, with 28 (4.2%) testing positive for syphilis (past or recent). Of the 28 who tested positive for syphilis, 24 (85.7%) tested positive in the laboratory, with 8/24 (33.3%) testing RPR positive. The proportion of men with recent/past syphilis infection in the laboratory was 43/609 (7.1%), while that with recent syphilis was 9/609 (1.5%). The median RPR titre levels among the nine who were RPR positive was 8 (IQR 1–16), with 5/9 men having high titres (≥ 8) suggestive of untreated infection. Two of the nine men who had recent syphilis were also HIV positive.

Compared to those who tested negative for syphilis on the rapid dual HIV/syphilis test; those who tested positive were older (median age 43 years vs 32 years, *P* < 0.001), more likely to be divorced or widowed (17.4% vs 4.1%, *P* = 0.004), less likely to have completed secondary education (69.6% vs 81%, *P* = 0.014) and more likely to be self-employed (26.1% vs 11.5%) or unemployed (47.8% vs 39.4%, *P* = 0.004).

### Performance of dual HIV/syphilis RDT compared to laboratory testing

Table 3 presents the performance of the RDT on site compared to centralised laboratory testing. Overall, there was good agreement between RDT and laboratory testing for both syphilis and HIV. However, there was sub-optimal sensitivity for HIV detection (90.9%; 95% CI 88.5% – 93.3%) and for detection of any syphilis (past or recent) (55.8%; 95% CI 51.9% – 59.8%). Sensitivity improved to 88.9% (95% CI 86.4% – 91.4%) for recent syphilis detection. Specificity for HIV detection was 99.2% (95% CI 98.5% – 100%), and for syphilis, 99.3% (95% CI 98.5% – 100%). Although the RDT missed 44% of men who had past syphilis infection, it was able to detect 8/9 men with recent syphilis infection. Online Appendix 1 Table 2 shows the performance of the assay by site of enrolment across the six sites.

**TABLE 3:** Performance of the dual HIV/syphilis testing with First Response HIV1+2/Syphilis Combo Card Test®.

Testing component	*N*	Sensitivity		Specificity		Positive predictive values		Negative predictive values		Agreement	
		Estimate	95% CI	Estimate	95% CI	Estimate	95% CI	Estimate	95% CI	Estimate	95% CI
HIV testing	544	90.9	88.5–93.3	99.2	98.5–100	83.3	80.2–86.5	99.6	99.1–100	99.9	97.6–99.5
Past and recent syphilis testing	609	55.8	51.9–59.8	99.3	98.6–100	85.7	82.9–88.5	96.7	95.3–8.1	96.2	94.4–97.5
Recent syphilis testing	609	88.9	86.4–91.4	96.7	95.2–98.1	28.6	25.0–32.2	99.8	99.5–100	96.6	94.8–97.7

CI, confidence interval.

### Post-test acceptability of rapid dual HIV/syphilis testing among men tested for HIV and syphilis on site

**FIGURE 2:**
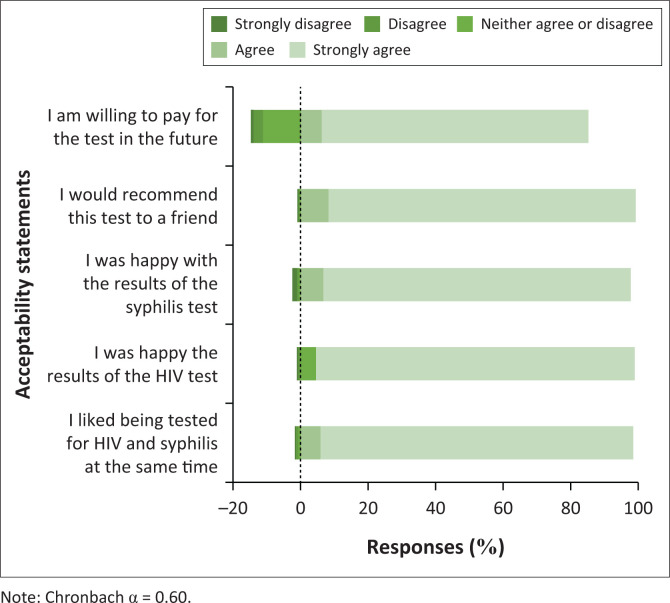
Post-test acceptability of dual HIV/syphilis testing (*n* = 605).

The majority of the 605 men tested using the dual HIV/syphilis rapid test had an overall positive experience (Figure 2). The overall median acceptability score was 25 (IQR: 25–25) out of a highest possible score of 25. Overall acceptability was 96.6% in contrast to 86.1% who were willing to pay for future testing with the dual HIV/syphilis test. Because all items on acceptability, except for willingness to pay, were scored highly and lacked variability in responses, willingness to pay was then used as a proxy for acceptability of rapid dual HIV/syphilis testing. Among men who were willing to pay for future testing and provided an amount they were willing to pay (472/605 [78.0%]), the median amount they were willing to pay was 50 ZAR (IQR: 25–100 ZAR).

Willingness to pay for the dual HIV/syphilis test was associated with being enrolled in Ekurhuleni district vs Tshwane (adjusted prevalence rate ratio [aPRR] 2.80 [95% CI: 2.09–3.77]) and being self-employed vs formally employed (aPRR 1.12 [95% CI: 1.02–1.22]), being a student/unable to work vs formally employed (aPRR 1.13 [95% CI: 1.02–1.25]), ever been paid to have sex (aPRR 1.08 [95% CI: 1.01–1.17]) and considering oneself to be at risk of HIV acquisition (aPRR 1.08 [95% CI: 1.02–1.14]) (Table 4). Age, marital status, risky sexual behaviours, knowledge of HIV status, and HIV or syphilis status on RDT were not associated with future willingness to pay for the dual HIV/syphilis test.

**TABLE 4:** Factors associated with willingness to pay for dual HIV/syphilis testing among who tested at VMMC sites.

Variable	Willing to pay for test (*n* = 605)			Univariable PRR		*P*	Multivariable PRR (*n* = 507)		*P*
	*N*	*n*	%	Estimate	95% CI		Estimate	95% CI	
**Age (years)**	-	-	-	-	-	0.467	-	-	0.341
≤ 24	120	104	86.7	1.03	0.95–1.12	-	1.04	0.96–1.11	-
> 24	485	408	84.1	1.00	-	-	1.00	-	-
**District**	-	-	-	-	-	< 0.001	-	-	< 0.001
Ekurhuleni	104	35	33.7	1.00	-	-	1.00	-	-
Tshwane	501	477	95.2	2.83	2.16–3.71	-	2.80	2.09–3.77	-
**South African-born**									
No	105	84	80.0	1.00	-	-	1.00	-	-
Yes	500	428	85.6	1.07	0.97–1.19	0.195	1.07	0.98–1.18	0.143
**Marital status**									
Never married	384	323	84.1	1.00	-	-	-	-	-
Married or living as married	185	158	85.4	1.02	0.94–1.09	0.438	-	-	-
Divorced or widowed	28	25	89.3	1.06	0.93–1.22	-	-	-	-
**Highest level of education completed**									
Primary	58	52	89.7	1.01	0.89–1.14	-	-	-	-
Secondary	185	158	83.5	0.94	0.85–1.04	-	-	-	-
Tertiary or other	62	55	88.7	1.00	-	-	-	-	-
**Employment status**									
Formally employed	221	178	80.5	-	-	-	1.00		-
Self-employed	72	64	88.9	1.10	0.99–1.22	0.064	1.12	1.02–1.22	0.018
Student or learner or unable to work	67	62	92.5	1.15	1.05–1.26	0.004	1.13	1.02–1.25	0.020
Unemployed or looking for work	234	198	84.6	1.05	0.97–1.14	0.254	1.03	0.96–1.10	0.402
**Ever paid for sex**									
No	508	432	85.0	1.00	-	-	-	-	-
Yes	92	75	81.5	0.96	0.86–1.06	0.426	-	-	-
**Ever been paid to have sex**									
No	554	465	83.9	1.00	-	-	1.00	-	-
Yes	23	22	95.0	1.14	1.04–1.25	0.007	1.08	1.01–1.17	0.030
**Sex under the influence of alcohol or drugs in the past 3 months**									
No	468	402	85.9	1.00	-	-	1.00	-	-
Yes	107	84	78.5	0.91	0.82–1.06	0.096	0.98	0.92–1.05	0.629
**Condom use at last sexual encounter**									
No	349	296	84.8	1.00	-	-	-	-	-
Yes	215	181	84.2	0.99	0.81–0.89	0.842	-	-	-
**Two or more sexual partners in the last 3 months**									
No	315	258	81.9	1.00	-	0.394	-	-	-
Yes	70	54	77.1	0.94	0.82–1.08	-	-	-	-
**Treated for an STI in the past 12 months**									
No	453	383	84.6	1.81	1.05–3.12	0.032	1.52	0.90–2.59	0.121
Yes	15	7	46.7	1.00	-	-	1.00	-	-
Missing	137	122	89.1	1.91	1.11–3.29	0.020	1.54	0.90–2.62	0.113
**Considers oneself at risk for HIV infection**									
No	461	382	82.9	1.00	-	-	1.00	-	-
Yes	119	109	90.8	1.09	1.02–1.18	0.012	1.08	1.02–1.14	0.008
**Know HIV status**									
No	146	132	90.4	1.08	1.02–1.17	0.014	0.99	0.94–1.04	0.708
Yes	430	357	83.0	-	-	-	1.00	-	-
**HIV positive (RDT)**									
No	579	490	84.6	1.00	-	-	-	-	-
Yes	26	22	84.6	1.00	0.85–1.18	0.999	-	-	-
**Syphilis positive (RDT)**									
No	580	490	84.5	1.00		-	-	-	-
Yes	25	22	88.0	1.04	0.90–1.21	0.592	-	-	-

CI, confidence interval; PRR, prevalence rate ratio; RDT, rapid diagnostic testing; STI, sexually transmitted infection.

## Discussion

We evaluated the acceptability, outcomes and performance of a dual HIV/syphilis test – First Response HIV1+2/Syphilis Combo Card Test – among men attending VMMC services at six public sector VMMC facilities located in two districts of Gauteng Province, South Africa, during June–October 2021. We found that dual HIV/syphilis testing was acceptable within VMMC services, and that recent syphilis positivity was higher than in the previous general population estimates. We also found that while dual HIV/syphilis test had sub-optimal sensitivity for both HIV and syphilis compared to centralised laboratory testing, syphilis detection was improved for men who had recent syphilis, with the syphilis component of the RDT able to identify most men with recent infection.

The acceptability of dual HIV/syphilis testing has been investigated among different populations in the past. Most studies that have evaluated acceptability of dual HIV/syphilis testing have done so among MSM and TGW, FSW and pregnant women attending antenatal care. In these settings, dual HIV/syphilis testing was found to be acceptable among clients and to increase access and coverage of testing while reducing the time to treatment among positives.^23,24,25,26^ In our study, dual HIV/syphilis testing was acceptable with high uptake, despite a lack of demand generation activities prior to the study. Dual HIV/syphilis testing maybe a useful addition to services already available on the VMMC platform. Given the older age and higher likelihood of being single or widowed among men who were syphilis positive on a rapid, dual HIV/syphilis test, providing this service may have the potential to attract older sexually active men at risk of STI and HIV acquisition to the platform. The potential impact of syphilis testing and other STI screening services on the demographic and behavioural profile of men coming in for VMMC-related services, and on demand for male circumcision, will need to be investigated in implementation studies.

The prevalence of recent syphilis was 1.5% and higher than the 0.9% estimated for men in the general population in 2017.^1^ The increase may be due to increasing syphilis transmission that is reflected in the increasing seroprevalence among pregnant women.^5^ Offering syphilis screening within VMMC services maybe an appropriate strategy to identify men with previously undiagnosed syphilis for treatment and partner tracing and treatment. The sub-optimal sensitivity of the RDT is a limitation of the testing and has been reported elsewhere; in field evaluations of dual HIV/syphilis testing, sensitivities of 47% – 96% have been reported.^23,24,25,27,28,29^ This sub-optimal sensitivity is mitigated by the test’s ability to detect most active or recent infections, infections that have the most potential for transmission if left untreated. South Africa is in the process of introducing dual HIV/syphilis testing for pregnant women and will procure kits that are prequalified by the WHO. There is need for the country to explore other populations at similar or higher risk of undiagnosed HIV or syphilis, and other healthcare platforms for delivering this service.

Our study described the acceptability, outcomes and performance of rapid dual HIV/syphilis testing among heterosexual cis-gender men, likely one of very few studies to do so. The study had a number of limitations. First, the study was conducted under routine VMMC settings using routine VMMC staff for enrolment and testing procedures. Second, our study was only able to achieve 62% of the desired sample size, largely as a result of disruptions to VMMC services due to coronavirus 2019 (COVID-19) related illness among staff and low volumes of VMMC visits from clients being fearful of COVID-19 infections. Third, there were some data quality issues with missing data and poor specimen collection and handling after enrolment. This was despite implementation of quality assurance activities in the study. These issues could have been due to staff being overburdened with enrolment procedures in addition to their core VMMC responsibilities. Despite these limitations, our study provided insights into dual HIV/syphilis testing as an additional service to include on the VMMC platform.
